# Neuromuscular Junction Abnormalities in Mitochondrial Disease: An Observational Cohort Study

**DOI:** 10.1212/CPJ.0000000000000795

**Published:** 2021-04

**Authors:** Luis P. Braz, Yi Shiau Ng, Gráinne S. Gorman, Andrew M. Schaefer, Robert McFarland, Robert W. Taylor, Doug M. Turnbull, Roger G. Whittaker

**Affiliations:** Department of Neurology (LPB), Centro Hospitalar Universitário de São João, Porto, Portugal; and Wellcome Centre for Mitochondrial Research (YSN, GSG, AMS, RM, RWT, DMT), Translational and Clinical Research Institute (RGW), Newcastle University, Newcastle upon Tyne, United Kingdom.

## Abstract

**Objective:**

To determine the prevalence of neuromuscular junction (NMJ) abnormalities in patients with mitochondrial disease.

**Methods:**

Eighty patients with genetically proven mitochondrial disease were recruited from a national center for mitochondrial disease in the United Kingdom. Participants underwent detailed clinical and neurophysiologic testing including single-fiber electromyography.

**Results:**

The overall prevalence of neuromuscular transmission defects was 25.6%. The highest prevalence was in patients with pathogenic dominant RRM2B variants (50%), but abnormalities were found in a wide range of mitochondrial genotypes. The presence of NMJ abnormalities was strongly associated with coexistent myopathy, but not with neuropathy. Furthermore, 15% of patients with NMJ abnormality had no evidence of either myopathy or neuropathy.

**Conclusions:**

NMJ transmission defects are common in mitochondrial disease. In some patients, NMJ dysfunction occurs in the absence of obvious pre- or post-synaptic pathology, suggesting that the NMJ may be specifically affected.

Fatigue is one of the most common symptoms in patients with mitochondrial disease.^1,2^ A cardinal feature of these disorders is multisystem involvement, and this fatigue may originate in 1 or several locations in the brain, spinal cord, peripheral nerve, and skeletal muscle.^3,4^

A prime candidate is the neuromuscular junction (NMJ). There are striking similarities between patients with some forms of mitochondrial disease and myasthenia gravis. Ptosis, ophthalmoplegia, and proximal muscle weakness are commonly described in both conditions, and several articles have described the difficulty of distinguishing between them on clinical grounds alone.^5–7^ The picture is further complicated by reports of abnormal neurophysiologic indices of NMJ dysfunction in patients with mitochondrial disease.^8–10^ However, these have been case reports or small series in patients in whom a diagnosis of mitochondrial disease was not always genetically proven. Furthermore, no attempt has been made to investigate the mechanism of the observed NMJ abnormalities. The prevalence of NMJ abnormalities in mitochondrial disease remains unclear, and it remains unknown whether these abnormalities are a direct consequence of mitochondrial dysfunction at the end plate or merely reflect NMJ damage from the neuropathy and myopathy commonly found in these patients.^11,12^

To address these questions, we present a large systematic study of NMJ function in a cohort of patients with genetically proven mitochondrial disease. This work provides evidence that NMJ dysfunction is independent of coexisting neuropathy and myopathy, raising the possibility that the NMJ may be specifically vulnerable to mitochondrial dysfunction.

## Methods

Eighty participants (mean age 49.5 years, range 18–81 years, 29.8% males) with a confirmed genetic diagnosis of mitochondrial disease were recruited from a specialized mitochondrial disease clinic held in the Highly Specialised Rare Mitochondrial Disorders of Adults and Children Service, Newcastle Hospitals National Health Service Foundation Trust. This is one of 3 nationally commissioned mitochondrial disease clinics in the United Kingdom. Referrals were either based on a clinical suspicion of neuromuscular system involvement or were performed as routine screening in accordance with the Newcastle Mitochondrial Disease Neuropathy Guideline.^13^

Participants were assessed clinically using the Newcastle Mitochondrial Disease Scale for Adults (NMDAS), a semiquantitative clinical rating scale designed for all forms of mitochondrial disease.^14^ Each subsection was scored between 0 and 5 and included assessments of exercise tolerance, gait, ptosis, external ophthalmoplegia, proximal weakness, and deep tendon reflexes. Blood was also taken for creatine kinase (CK) level and glycosylated hemoglobin (HbA1c).

All neurophysiology studies were performed and reviewed by a consultant neurophysiologist (R.G.W.). Nerve conduction studies were performed using surface electrodes (Natus Medical) on a Keypoint electromyography (EMG) machine (Dantec). For motor studies, the median, ulnar, common peroneal, and tibial nerves were studied; for sensory studies, the median, ulnar, sural, and superficial peroneal nerves were assessed. Results were compared with published reference data and classified as normal, motor-sensory axonal polyneuropathy, sensory axonal neuropathy, sensory neuronopathy, and motor-sensory demyelinating neuropathy.

EMG was performed using a 25-G concentric needle (Natus Medical) on the muscle(s) studied in single-fiber electromyography (SFEMG): extensor digitorum communis (EDC) and/or orbicularis oculi (OO). In addition, EMG was performed on at least 2 other muscles (deltoid, biceps brachii, vastus lateralis, and tibialis anterior). Analysis of spontaneous activity at rest, individual motor unit potentials, and of interference pattern was made, and the results classified as normal, neurogenic, or myopathic.

Repetitive nerve stimulation was performed in the first 33 participants (42.3%) and was normal in all. Patients found this to be very uncomfortable, and for this reason, we limited the examination to the more sensitive single-fiber EMG in the remaining participants.

SFEMG was performed on the EDC and/or OO muscles using a 25-G facial concentric needle (bandpass 1–10 kHz). SFEMG was not possible in 2 patients because of severe myoclonus or tremor. SFEMG was performed on the EDC muscle in all of the remaining 78 patients and in the OO muscle in 15 patients. The proportion of pairs with increased jitter and/or blocking fibers was recorded, and the study was considered abnormal when ≥10% of fiber pairs showed increased jitter or blocking.^15^

Statistical analysis was performed using IBM SPSS Statistics V24. Parametric data were compared between groups using the unpaired Student *t* test, and nonparametric data were compared using the Chi statistic.

### Standard Protocol Approvals, Registrations, and Patient Consents

The study was approved by Newcastle and North Tyneside Local Research Ethics Committee. All patients gave written consent before study enrollment.

### Data Availability

Anonymized study data will be made available on request.

## Results

### Cohort Characteristics

The most common specific genotypes were the m.3243A>G pathogenic variant (30.6% of patients), followed by single, large-scale mitochondrial DNA (mtDNA) rearrangements (13.9%) and then pathogenic variants in mendelian-inherited mitochondrial genes encoding proteins involved in mtDNA maintenance: twinkle (11.1%), OPA1 (9.7%), polymerase gamma (8.3%), and RRM2B (6.9%). The remaining rarer genotypes each comprised fewer than 5% of the total cohort (table).

**Table:**
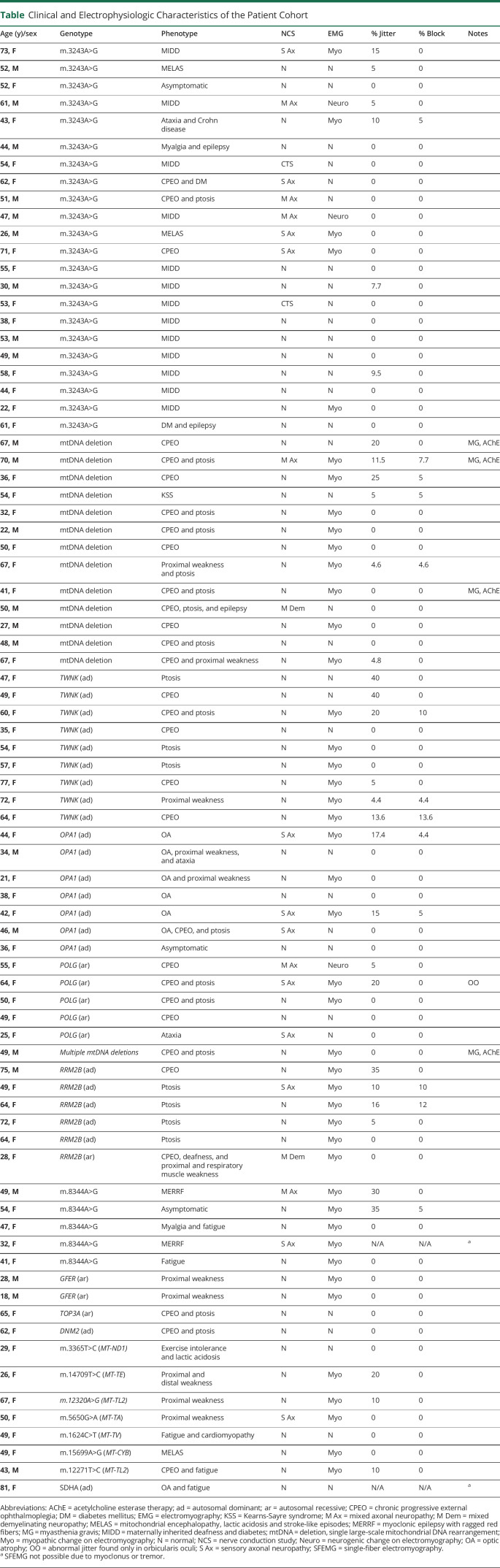
Clinical and Electrophysiologic Characteristics of the Patient Cohort

The most common clinical manifestations attributable to mitochondrial disease were progressive external ophthalmoplegia and ptosis (59% of patients), muscle weakness (52.6%), ataxia (38.5%), prominent fatigue/myalgia (37.2%), sensorineural hearing loss (30.8%), diabetes mellitus (24.4%), and cardiomyopathy (17.9%). Most participants exhibited a composite phenotype of multiple symptoms and signs.

### Prevalence of NMJ Abnormalities

SFEMG was not possible in 2 participants because of severe myoclonus or tremor. Twenty of 78 of the remaining participants (25.6%) had evidence of NMJ dysfunction. The highest prevalence of NMJ abnormalities was found in patients with pathogenic RRM2B variants (3 of 6 = 50%), followed by TWNK (4 of 9 = 33.3%), m.8344A>G (2 of 5 = 40%), OPA1 (2 of 7 = 28.6%), single, large-scale mtDNA rearrangements (3 of 13 = 23%), POLG (1 of 6 = 16.7%), and m.3243A>G (2 of 22 = 9.1%). The remaining genotypes comprised fewer than 5 subjects each, making estimation of the prevalence of NMJ dysfunction unreliable.

The highest percentage of both jittering and blocking fiber pairs was found in participants with the Twinkle mutation (up to 40% and 13.6%, respectively). No association was found between severity of jitter or blocking and overall disease severity assessed using the NMDAS scale, CK, or HbA1c level.

### Correlation With Coexisting Neuromuscular Disorders

Neurophysiologic evidence of neuropathy was detected in 18 of 78 participants (23%); in 11 of these, only sensory fibers were involved; in 6, a mixed motor and sensory axonal neuropathy was found; and in 1 patient, a mixed demyelinating neuropathy was found. Two participants had evidence of carpal tunnel syndrome but not of a polyneuropathy. Myopathy was found in 41 of 78 participants (52.6%). Ten participants had both neuropathy and myopathy; in 8 of these, the neuropathy affected sensory fibers only.

Treating neuropathy, myopathy, and NMJ dysfunction as independent conditions, we compared the frequency of neuropathy and myopathy in participants with and without NMJ dysfunction.

Treating neuropathy, myopathy, and NMJ dysfunction as independent conditions, we compared the frequency of neuropathy and myopathy in participants with and without NMJ dysfunction. The frequency of neuropathy was similar in participants with and without NMJ dysfunction (Chi statistic 0.97 *p* = 0.325). Furthermore, no participants with neuropathy alone had evidence of NMJ dysfunction. In contrast, the frequency of myopathy differed significantly between groups, being higher in the group with NMJ dysfunction (Chi statistic 7.16, *p* = 0.0074). NMJ dysfunction was also found in 9 patients with myopathy alone.

We found no difference in the overall NMDAS score, exercise tolerance, gait, ptosis, external ophthalmoplegia, CK level, or HbA1c level between participants with and without NMJ dysfunction.

Three of the 20 participants (15%) with NMJ abnormality had no neurophysiologic evidence of either neuropathy or myopathy (1 patient with a single, large-scale mtDNA rearrangement and 2 patients with dominant TWNK variants). The mean percentage of abnormal fiber pairs in participants with neither myopathy nor neuropathy was higher than in participants with myopathy alone or both myopathy and neuropathy (33.3% in participants with neither vs 20.5% myopathy alone vs 17.0% in both).

## Discussion

We present a large systematic study of NMJ function in a cohort of patients with genetically proven mitochondrial disease. We find that 20 of 78 (25.6%) participants included in the study population had abnormal NMJ transmission, with jitter values similar to patients with myasthenia gravis. NMJ abnormalities were seen in a wide range of mitochondrial genetic defects including nuclear gene defects, single large-scale mtDNA rearrangements, and mtDNA point mutations.

Our cohort was recruited from one of 3 national referral clinics for mitochondrial disease, and all had genetically proven mitochondrial disease. The spread of genotypes is broadly similar to published population-based studies in that the pathogenic m.3243A>G variant and single, large-scale mtDNA deletions represent the 2 most common mitochondrial genotypes presenting with multisystem disease.^16^ However, our cohort was referred for neurophysiologic testing based on a clinical suspicion of coexisting neuromuscular disease. Consequently, it is likely that the prevalence of NMJ dysfunction in our cohort overestimates that in all patients with mitochondrial disease, which include a considerable number of asymptomatic carriers. However, to perform such detailed and time-consuming neurophysiologic testing in asymptomatic carriers presents significant logistical and ethical barriers.

Mitochondria are abundant in both the presynaptic motor nerve terminals and the postsynaptic junctional folds.^17^ Given the key role of mitochondria in vesicle release and recycling,^18^ it is perhaps surprising that we find no association between the presence of neuropathy and NMJ dysfunction. This may be because the majority of patients had a pure sensory neuropathy, which would not be expected to affect the NMJ. In contrast, we find a strong association between NMJ dysfunction and the presence of myopathy across all genotypes. This may be coincidental, given that both NMJ dysfunction and myopathy are common features in mitochondrial disease. However, abnormal jitter and blocking is described in several myopathies, including myotonic dystrophy type 1,^19^ polymyositis,^20^ and inclusion body myositis.^21^ The mechanism remains unclear, although destruction of the postsynaptic folds has been described in a rodent model of Duchenne muscular dystrophy.^22^ Whether similar changes occur in mitochondrial disease is unknown, and to our knowledge, no ultrastructural studies of NMJ morphology in mitochondrial disease have been performed.

Arguing against a causal relationship is the observation that in 15% of patients with NMJ dysfunction, no evidence was found of either myopathy or neuropathy. Of interest, the percentage of fibers with increased jitter was higher in this group than in those with myopathy alone or both myopathy and neuropathy. These patients with a primary defect of neuromuscular transmission in what are presumably structurally normal NMJs may be a particularly suitable group to target with therapies that boost NMJ transmission.

Arguing against a causal relationship is the observation that in 15% of patients with NMJ dysfunction, no evidence was found of either myopathy or neuropathy.

Unfortunately, we found no simple means of identifying these patients before neurophysiologic testing. Patients with significant NMJ dysfunction did not differ in their disease severity as assessed using the NMDAS scale or in any of the relevant subsections. There was also no difference on routine blood tests (CK and HbA1c level). The only predictor of NMJ dysfunction was electromyographic evidence of myopathy; however, relying on this alone would fail to identify those patients with a “pure” NMJ disorder. This means that at present, the only means of reliably detecting NMJ dysfunction is with detailed neurophysiology including single-fiber EMG. The time taken to perform this technique, the need for specialist expertise, and the limited availability in some countries limit the applicability of these findings. Nevertheless, a case can be made that patients being seen in a specialist center in which these are available should be considered for SFEMG at least once.

The distinction between genetically proven mitochondrial disease and autoimmune myasthenia gravis (particularly ocular myasthenia) is not always obvious on clinical grounds alone, and our data show that SFEMG abnormalities can be seen in both patient populations. Patients with autoimmune myasthenia gravis typically show marked fluctuation in symptom severity, often have bulbar symptoms early in the disease, and an association with other autoimmune diseases.^23^ In contrast, patients with mitochondrial disease typically have limited symptom fluctuation, develop bulbar symptoms late on, and have a pattern of multisystem disease including other neurologic symptoms such as seizures and ataxia.^24^ Autoantibody testing (including antibodies to the acetylcholine receptor, muscle specific kinase, LRP4, and agrin) can help confirm an autoimmune etiology, but up to 15% of patients are seronegative for known autoantibodies. Perhaps the most important point is that mitochondrial disease should be considered in patients with suspected autoimmune myasthenia gravis who fail to respond to immunosuppression.

**TAKE-HOME POINTS**

→ NMJ abnormalities are common in mitochondrial disease.
→ Abnormalities can occur in the absence of coexisting neuropathy or myopathy, suggesting specific involvement of the NMJ in mitochondrial disease.
→ Whether modulators of neuromuscular transmission have a role in treating these patients is worthy of further study.
